# The impact of follicle-flushing during oocyte collection on embryo development of in-vitro fertilization

**DOI:** 10.1186/s12958-019-0540-5

**Published:** 2019-12-05

**Authors:** Li-hua Zhu, Xiao-bei Ni, Fei Lin, Zhi-peng Xu, Jun-shun Fang, Ning-yuan Zhang

**Affiliations:** Reproductive Medical Center, Drum Tower Hospital Affiliated with Nanjing University Medical College, Zhongshan Road 321#, 210008 Nanjing, People’s Republic of China

**Keywords:** Oocyte retrieval difficulty, Number of recovered oocytes, Embryo development potential, Blastulation

## Abstract

**Background:**

To evaluate the impact of follicle-flushing during oocyte collection on embryo development potential retrospectively.

**Methods:**

A total of 1714 cases, including 133 who experienced retrieval difficulty (repeated follicle-flushing) on the day of oocyte retrieval (difficulty group) and the control 1581 cases (control group), were assessed in this retrospective study. The number of oocytes recovered, two pro-nuclei fertilization (2PN-fertilization), day 3 good-quality embryo and day 5/6 blastocyst utilization rates were compared between the difficulty group and control group correspondingly. Embryo implantation, clinical pregnancy and neonatal outcomes were further analyzed between the two groups in the fresh day^− 3^ embryo transfer cycles.

**Results:**

The number of oocytes recovered in the difficulty group (9.08 ± 4.65) were significantly reduced compared with the control group (12.13 ± 5.27),*P* < 0.001; The 2PN-fertilization, day 3 good-quality embryo and blastocyst utilization rates were significantly lower in the difficulty group compared with controls (71.7% vs. 75.7%; 52.7% vs. 56.5%; 31.9% vs. 37.0%, all *P* < 0.05). Embryo implantation in the difficulty group was 53.2%, which was lower than the control value of 58.7%, although not reaching statistical significance. The rate of fresh embryo transfer cycles in the difficulty group was lower than normal ones (51.88% vs. 61.99%, *P* = 0.026). The pregnancy and live birth rates were similar between the two groups. But the rate of spontaneous miscarriages of the difficulty group was higher than the control group, although not reaching statistical significance. The neonatal outcomes had no statistical difference between the two groups.

**Conclusions:**

Oocyte retrieval difficulty, which include repeated flushing and the corresponded extending time required for oocyte recovery, significantly reduced day 3 good-quality embryo and blastocyst utilization rates of these patients. But the live birth rate had no difference between the difficulty group and the normal ones.

## Background

Transvaginal ultrasound-guided oocyte retrieval is a technique in which under ultrasound guidance, a needle is inserted through the posterior fornix to the ovary, and a mature ovum is retrieved for in-vitro fertilization and embryo culture [1, 2]. This method has become a routine procedure for oocyte retrieval in human assisted reproduction, and is considered as an effective, safe, and required step for in-vitro fertilization and embryo transplantation (IVF-ET) [3–5]. It affects oocyte recovery number and oocytes quality, as well as the outcome of embryos growth [3, 6]. On the day of oocyte retrieval, we have encountered cases that no oocytes were recovered, regardless of many matured follicles; and cases that obtained a scant number of oocytes from numerous matured follicles. These patients may experience aspiration difficulty, even facing a situation that the operative time is too long and most of their follicles are flushed repeatedly [7]. High flushing pressure might cause early rupture of the follicular wall, which results in oocytes damage. In this retrospective study, 133 patients who experienced retrieval difficulty on the day of oocyte collection were assessed. The oocyte development potential, the clinical and the neonatal outcomes were analyzed in the article.

## Methods

### Study design

This retrospective study was approved by the Medical Ethics Committee of Drum Tower Hospital Affiliated with Nanjing University Medical College. All patients provided signed informed consent for IVF-ET treatment in 2017.

This retrospective study included women with regular menses who were undergoing their first cycle of in vitro fertilization (IVF) or intracytoplasmic sperm injection (ICSI) due to tubal factors, male factors, or both. Tubal factors included unilateral or bilateral tubal occlusion, peritubal adhesion, unilateral or bilateral salpingectomy, or tubal ligation. Male-factor infertility included oligospermia, asthenospermia, or obstructive azoospermia. Eligible women were 22 to 35 years old, had a normal menstrual cycle (defined as a spontaneous cycle length of 21 to 35 days), and a duration of infertility of more than 1 year. Women with a history of unilateral oophorectomy, recurrent spontaneous abortion, diagnosis of the polycystic ovary syndrome, or uterine abnormality (e.g., adenomyosis, mullerian duct anomaly, endometriosis, submucous myoma, intra- uterine adhesion, or scarred uterus) were excluded. Women were also excluded if they had renal disease, abnormal renal function, history of deep venous thrombosis, severe anemia, pulmonary embolus, or cerebrovascular accident. All the couples were screened with the use of karyotyping, and those with an abnormal karyotype were excluded.

## Methods

Each follicle with a diameter of > 10 mm was aspirated. The tip of the needle was directed to the centre of the follicle and the aspirate was considered complete when the follicle appeared to have completely collapsed on the ultrasound scan. This initial aspirate was handed to the embryologist, if an oocyte was not found, the dead space in the collecting system (needle and attached tubing) was then flushed with 2 ml culture medium until the first drop of medium was seen. If an oocyte was also not found, the follicle was flushed up to a maximum of six times. All of the recovered oocytes were flushed at least twice of a patient was considered oocyte retrieval difficulty [4, 7, 8].

This study involved 133 patients who presented to our center for the first oocyte retrieval cycle, and experienced retrieval difficulty on the day of oocyte aspiration. The control group included 1581 patients who had successful oocyte retrieval in our center. Age, Body-mass index, amounts of oocytes recovered, embryo development potential and the clinical outcome were compared between the two groups. Furthermore, based on different fertilization methods, patients receiving IVF and ICSI were individually compared for oocytes recovered, embryo development potential, and the clinical outcomes.

Ovulation induction and fertilization: Controlled ovarian hyperstimulation (COH) was performed according to the standard protocols of our center. All patients underwent COH with exogenous gonadotrophins used the following protocol: long GnRH agonist protocol, in which the agonist was started in the mid luteal phase of the preceding menstrual cycle, adding gonadotropins on the second day after menstrual bleeding. And ovulation was triggered when follicles reached > 17 mm in diameter, using 250 μg of human chorionic gonadotropin (hCG) (Ovitrelle®, Merck Serono, Italy). On the day of operation, routine IVF or ICSI was performed based on sperm quality.

Embryo culture: For IVF, insemination was performed after 4–5 h of culturing the retrieved oocytes in IVF-30 medium (G-IVF, 10135, Vitro-Iife, Sweden) supplemented with 10% synthetic serum substitute (Irvine Scientific, Santa Ana, CA); fertilization was confirmed by identification of pronuclei 16 h after insemination. All embryos were transferred into G1 medium (G^− 1^, 10,127, Vitro-life, Sweden) supplemented with 10% synthetic serum substitute and evaluated using the criteria of the Istanbul consensus [9]. For ICSI, sperm injection was performed according to the standard ICSI protocol, and fertilization was confirmed by identification of pronuclei 16 h after insemination. All embryos were transferred into G1 medium supplemented with 10% synthetic serum substitute and were evaluated using the criteria of the Istanbul consensus [9]. Day 4 to day 6 stage embryos were cultured in G2 medium (G–2, 10,131, Vitro-life, Sweden) supplemented with 10% synthetic serum substitute and embryos were evaluated using the criteria of the Istanbul consensus [10] .

Determination and monitoring of pregnancy: After day 3 fresh cleavage stage embryos transfer, blood or urine human chorionic gonadotropin (hCG) levels were measured at day 14 after embryos transfer. The patients with positive results underwent B-ultrasound examination 28 days later, and diagnosis criteria for pregnancy were the presence of gestational sac and primitive cardiac pulsation.

### Statistical analysis

Statistical analysis was performed with the SPSS 23.0 software. An independent samples t-test was used to compare the baseline characteristics of the participants and the means of the number of oocytes recovered. The χ^2^-test was used to compare 2PN-fertilization, cleavage, day 3 good-quality embryo, blastulation, blastocyst utilization, day 5 blastocyst utilization, day 6 blastocyst utilization, embryo implantation and clinical pregnancy rates. Measurement data are mean ± SD or %. Statistical significance was defined at *P* < 0.05.

## Results

### Oocyte retrieval and embryo development outcomes

The baseline characteristics of the total 1714 trial participants (Table 1) were similar in the oocyte retrieval difficulty group and the control group.

**Table 1:** Characteristics of the Participants at Baseline

	Difficulty group	Control	*P* Value
	(*N* = 133)	(*N* = 1581)	
Age---yr	28.99 ± 3.14	28.98 ± 3.10	0.99
Body-mass index	20.82 ± 1.32	20.89 ± 1.20	0.52
Antral Follicle Count (AFC)	17.50 ± 6.18	16.81 ± 5.64	0.50
Duration of infertility (year)	3.56 ± 2.51	3.44 ± 2.67	0.79
Type of infertility			
Primary	81	980	
Secondary	52	601	
Laboratory tests			
Follicle-stimulating hormone---IU/liter	7.66 ± 3.10	7.81 ± 2.74	0.67
Luteinizing hormone---IU/liter	6.79 ± 4.87	6.14 ± 3.98	0.24
Estradiol---pg/ml	48.45 ± 36.88	45.20 ± 77.68	0.68
Total testosterone---ng/ml	1.30 ± 5.88	1.48 ± 6.74	0.83

Note: Values are mean (±SD) unless otherwise indicated

In Table 2, compared with the control group, the oocyte retrieval difficulty group had a significantly lower amounts of oocytes recovered, and a decreased 2PN-fertilization rate as well as the decreased day 3 good-quality embryo, and blastocyst utilization (especially day 5 blastocyst utilization) rates.

**Table 2:** Comparison of clinical parameters between the oocyte retrieval difficulty group and the control group

	Difficulty group	Control	*P* Value
No. of cycles	133	1581	
Total gonadotrophins dose	1748.39 ± 654.12	1821.57 ± 785.23	0.79
E2 (pg/ml) in hCG Day	3444.23 ± 2224.30	3451.94 ± 1663.19	0.99
Progesterone(ng/ml) in hCG Day	0.95 ± 0.79	0.89 ± 0.55	0.59
Duration of COH	10.66 ± 3.14	9.98 ± 2.47	0.149
No. of follicles aspirated	12.17 ± 4.92	12.46 ± 4.53	
No. of recovered oocytes	9.08 ± 4.65	12.13 ± 5.27	*P* < 0.001
2PN-fertilization rate	800/1116 (71.7%)	13,290/17565 (75.7%)	0.003
Cleavage rate	774/800 (96.8%)	12,864/13290 (96.8%)	0.93
Day 3 good-quality embryo rate	408/774 (52.7%)	7271/12864 (56.5%)	0.04
Blastulation rate	203/423 (48.0%)	4050/7664 (52.8%)	0.06
Blastocyst utilization rate	135/423 (31.9%)	2838/7664 (37.0%)	0.03
Day 5 blastocyst utilization rate	90/423 (21.3%)	2004/7664 (26.1%)	0.02
Day 6 blastocyst utilization rate	45/333 (13.5%)	834/5660 (14.7%)	0.58

Note: Values are mean (±SD) unless otherwise indicated

### Oocyte retrieval and embryo development outcomes between the two groups by different fertilization types

According to different fertilization types, the patients were further divided into IVF and ICSI subgroups. 2PN-fertilization rate and embryo development potential were compared between the retrieval difficulty group and the control cases (Table 3).

**Table 3:** Comparison of clinical parameters between the oocyte retrieval difficulty group and the control group by IVF or ICSI

	Difficulty group	Control	*P* Value
Insemination methods			
IVF			
No. of cycles	98	1180	
Age---yr	29 ± 3.06	29 ± 3.06	0.99
No. of retrieved oocytes	9.49 ± 4.70	12.08 ± 5.29	*P* < 0.001
2PN-fertilization rate	655/930 (70.4%)	10,584/14258 (74.2%)	0.01
Day3 good-quality embryo rate	346/634 (54.6%)	5740/10245 (56.0%)	0.48
Blastulation rate	177/342 (51.8%)	3269/5907 (55.3%)	0.20
Blastocyst utilization rate	122/342 (35.7%)	2297/5907 (38.9%)	0.25
Day5 blastocyst utilization rate	86/342 (25.1%)	1725/5907 (29.2%)	0.11
Day 6 blastocyst utilization rate	36/256 (14.1%)	572/4182 (13.7%)	0.85
ICSI			
No. of cycles	35	401	
Age---yr	28.94 ± 3.40	28.90 ± 3.20	0.93
No. of retrieved oocytes	7.94 ± 4.35	12.25 ± 5.20	*P* < 0.001
No. of MII stage oocytes	5.31 ± 4.34	8.25 ± 5.14	0.001
2PN-fertilization rate	145/186 (78%)	2706/3307 (81.8%)	0.19
Day 3 good-quality embryo rate	62/140 (44.3%)	1531/2619 (58.5%)	0.001
Blastulation rate	26/81 (32.1%)	781/1757 (44.5%)	0.03
Blastocyst utilization rate	13/81 (16.0%)	541/1757 (30.8%)	0.005
Day 5 blastocyst utilization rate	4/81 (4.9%)	279/1757 (15.9%)	0.004
Day6 blastocyst utilization rate	9/77 (11.7%)	262/1216 (21.5%)	0.043

Note: Values are mean (±SD) unless otherwise indicated; IVF, in vitro fertilization; ICSI, intra-cytoplasmic sperm injection

In individuals receiving IVF, the difficulty group showed significantly less oocytes recovered and reduced rate of 2PN-fertilization. The difficulty group had a day 3 good-quality embryo rate of 54.6% and a day 5 blastocyst utilization rate of 25.1%, which were lower than the control group (56.0 and 29.2% respectively).

In cases receiving ICSI, the difficulty group showed significantly reduced amounts of MII stage oocytes, lower rates of day 3 good-quality embryo and blastocyst utilization rates (including day 5 and day 6).

### Clinical outcomes

A total of 69 and 980 cases in the difficulty and control groups received fresh day 3 embryo transfer. Embryo transfer cycles / oocyte retrieval cycles in the difficulty group was 51.88% which was lower than the control (61.99%), *P* = 0.026; the average number of transfered embryos were 1.80 ± 0.41 and 1.82 ± 0.38, respectively. The rate of embryo implantation in the difficulty group was 53.2%, which was lower than that of controls (58.7%), although the difference was not significant. The clinical pregnancy, live birth and the early miscarriage rates between the two groups had no statistical significance. (Table 4).

**Table 4:** Clinical outcomes between the oocyte retrieval difficulty group and the control group

	Difficulty group	control	*P* Value
Embryo transfer cycles	69	980	
Fresh embryo transfer cycles/oocyte retrieval cycles	69/133	980/1581	0.026
(%)	(51.88%)	(61.99%)	
Cycles having extra embryo cryopreservation	53/69	941/980	0.00
(%)	(76.81%)	(96.02%)	
Mean embryos transferred	1.80 ± 0.41	1.82 ± 0.38	
Endometrial thickness(mm) in ET day	9.80 ± 1.70	9.81 ± 1.53	0.959
Embryo implantation rate	66/124 (53.2%)	1046/1782(58.7%)	0232
Pregnancy rate	52/69 (75.4%)	715/980 (73.0%)	0.663
Spontaneous miscarriages	3	29	
(% per pregnancy, before 12 weeks)	(5.8)	(4.1)	0.551
Spontaneous miscarriages	3	16	
(% per pregnancy, after 12 weeks)	(5.8)	(2.2)	0.114
live birth rate	46/69(66.7%)	670/980(68.4%)	0.769

Note: Values are mean (±SD) unless otherwise indicated

### Neonatal outcomes

Neonatal outcomes are presented in Table 5. Fifty nine and 905 babies were born from oocyte retrieval difficulty group and the control group. The median gestational ages were 38.22 and 37.62 weeks. Preterm birth (32–37 weeks) occurred in 25.4 and 27.3% of the oocyte retrieval difficulty group and the control group (P>0.05). The median birthweight of babies were 2934.48 g and 2851.99 g of the two groups. No significant difference was found for the rate of birthweight (g) < 2500 of the live birth between the two groups.

**Table 5:** Neonatal outcomes between the oocyte retrieval difficulty group and the control group

	Difficulty group	control	*P* Value
Pregnancies	59	905	
Gestational age (weeks)	38.22 ± 3.54	37.62 ± 2.83	
Gestational age (weeks) <37w	15(25.4%)	247(27.3%)	0.755
Birthweight (g)	2934.48 ± 825.81	2851.99 ± 625.79	
Birthweight (g) <2500	12(20.3%)	222(24.5%)	0.467

Note: Values are mean (±SD) unless otherwise indicated

## Discussion

In vitro fertilization and embryo transfer, refers to the procedure that stimulates follicular development within the natural period or by gonadotropin administration, and then retrieves the mature ovum from the ovary for in vitro embryo culture and embryo transplantation [11–14]. The first step of IVF-ET is to aspirate the matured oocytes from the ovary [15, 16]. However, some patients may encounter difficulty on the day of oocyte collection. Among them, some may show failed oocyte retrieval, with consequently no embryo for transplantation [17]. Others, after repeated flushing, could obtain oocytes but a scant number of embryos suitable for transplantation [8]. This study retrospectively reviewed 133 cases who experienced difficulty on the day of oocyte collection, assessed the number of oocytes retrievaled, embryo development, clinical and neonatal outcomes.

It is generally admitted that the ovum can only worsen after in vitro handling [18, 19]. Many factors, such as air quality, light, pH, including in vitro operation, can cause a decrease in oocytes quality. The time taken for oocytes recovery and the difficulty for ovum retrieval are also important factors affecting oocytes quality [20, 21].

The current study included patients who experienced retrieval difficulty on the day of oocyte collection in 2017, alongside those with successful oocyte retrieval (control group), compared embryo development, the outcome of clinical pregnancy and neonatal outcomes between the two groups. We also compared embryo development in patients with different methods of fertilization, to analyze if the process of oocyte recovery might influence oocytes quality and embryo development potential. In cases receiving IVF, the difficulty group showed reduced rates of 2PN fertilization and day 5 blastocyst utilization compared with controls. In cases receiving ICSI, the difficulty group showed decreased number of MII stage oocytes, lower rates of day 3 good-quality embryo and day 5 blastocyst utilization.

In in vitro fertilization, good-quality embryos are important to successful embryo implantation [22, 23]. So, increasing the amount of retrieved oocytes is an effective way to improve the count of high-quality embryos [17, 23–25]. During ovum collection, some patients, due to various reasons, may experience retrieval difficulty, and finally obtain oocytes after repeated flushing. However, the repeated flushing pressure might cause early rupture of the follicular wall, which results in oocyte damage. Sometimes, oocyte damage could not be observed via the cumulus oocyte complexes, although it has already caused certain functional changes to the oocytes [26]. As reported previously, increased pressure during ovum retrieval would cause parthenogenetic activation in the oocyte, suggesting that pressure could change the physiological functions of the ovum [2]. In addition, repeated pressure on the oocytes could promote the formation of sterile oocytes, but the cumulus cells are critical for in vitro maturation, particularly cytoplasm maturation in oocytes [27]. In spite of nuclear maturation, oocytes without cumulus cells have limited developmental potential after fertilization, because cytoplasm maturation has been affected significantly. These embryos hardly develop into blastocysts, which yields very low clinical pregnancy rate [18, 28]. On the other hand, oocytes retrieval difficulty may indicate that the patient might have more abnormal oocytes than the control group, some abnormal oocytes may reflect genetic abnormalities and the development potential of these oocytes were low [29].

As shown in this article, the difficulty group had significantly reduced oocyte utilization rate compared with controls. The retrieval difficulty group showed lower embryo implantation, cycles having embryos to transfer and clinical pregnancy rates compared with controls.

It would be good to know the cumulative delivery rate across all transfers per initiated cycle to evaluate the overall quality of the oocytes retrieved, but not all the patients having their frozen embryo transfer until now. So, we did not show this result in this study.

All in all, in patients having retrieval difficulty, how to improve the number of oocytes collected and especially improve the quality of the oocytes remains a challenge worth more widespread investigation.

## Conclusion

In assisted reproduction, high-quality ovum is the basic and primary requirement for successful IVF [25]. Increasing the number of retrieved oocytes is an effective way to improve the number of transplantable or even high-quality embryos. Repeated flushing and extended time required for oocyte recovery during the process of oocyte retrieval, significantly reduced oocyte and embryo development potential.

## Data Availability

The datasets used and/or analysed during the current study are available from the corresponding author on reasonable request.

## Abbreviations

2PN: two pronuclei
hCG: human chorionic gonadotropin
ICSI: Intracytoplasmic sperm injection
IVF: In vitro fertilization
IVF-ET: In vitro fertilization and embryo transplantation
